# Australian Public and Smallpox

**DOI:** 10.3201/eid1111.041129

**Published:** 2005-11

**Authors:** David N. Durrheim, Reinhold Muller, Vicki Saunders, Rick Speare, John B. Lowe

**Affiliations:** * Hunter New England Population Health, Newcastle, New South Wales, Australia; † Newcastle University, Newcastle, New South Wales, Australia; ‡ James Cook University, Townsville, Queensland, Australia; § University of Iowa, Iowa City, Iowa, USA

**Keywords:** Bioterrorism, Australia, smallpox, dispatch

## Abstract

A national survey of 1,001 Australians found that most were concerned about a bioterrorist attack and were ill-informed about smallpox prevention and response. Since general practitioners were commonly identified as the initial point of care, they should become a focus of bioterrorism response planning in Australia.

Australia has identified protection against bioterrorism as a national research priority, and the Commonwealth Chief Medical Officer emphasizes the "need to be prepared for a bioterrorism incident" (*1*). Preparations have focused on central public health surveillance, with little attention to public understanding of bioterrorism (*2*).

Smallpox and anthrax are considered potential bioterrorism agents (*1**,**3**,**4*). Although smallpox response guidelines have been prepared for Australia (http://www.health.gov.au/internet/wcms/Publishing.nsf/Content/health-pubhlth-publicat-

document-metadata-smallpox.htm), the level of community awareness of these recommendations is unknown. In the event of an attack, the response of the public will be based on persons' current knowledge, beliefs, and patterned behavior (*5**,**6*). We conducted a cross-sectional national survey in Australia to assess knowledge and views about smallpox, vaccination, and other mitigation strategies.

## The Study

A list of private telephone numbers was randomly selected for each of Australia's 8 states and territories that was proportional to their contribution to the adult population. Participants were recruited to provide a sample size of 1,000, which allowed a precision of 2%–3% when calculating a 95% confidence interval of a dichotomous variable with a base proportion ranging from 10% to 50%.

Eight experienced telephone interviewers conducted the survey during July 2004. Repeat calls were conducted when persons indicated interest in participating but were unable to do so during the initial contact. The questionnaire was administered upon agreement to participate, after introducing the survey's purpose, providing a guarantee of confidentiality, and giving reassurance of freedom to withdraw consent.

The questionnaire was pretested for length and comprehensibility in a pilot study during May 2004. The final instrument took 10–15 minutes to administer and contained 22 questions. Data were analyzed by using SPSS for Windows version 11 (SPSS Inc., Chicago, IL, USA). Ethical approval was granted by the human ethics subcommittee at James Cook University (Nr. H1745).

A total of 1,001 Australian adults completed the survey. Two hundred thirteen were excluded (38 children, 91 adults with limited English ability, 9 incoherent adults, and 75 adults contacted at their workplace), and 582 refused to participate (response rate 63.2%). Respondents were geographically representative of the Australian population.

Respondents' ages were normally distributed (mean 52.2 years, standard deviation 17 years) and 62.8% (629) were female. Most (58.6%, 587) lived in cities, which reflected the situation in Australia, where 66.3% of the population live in urban areas. The level of education of respondents reflected that of the Australian population.

Concern about the risk of a bioterrorist attack in Australia was perceived as high by 182 (18.2%), medium by 392 (39.2%), low by 339 (33.9%), and nonexistent by 14 (1.4%); 72 (7.2%) did not know and 2 (0.2%) did not answer. Logistic regression modeling showed that age was the only demographic feature significantly associated with perceiving high risk of a bioterrorist attack (compared with low, medium, or none), with an odds ratio of 1.016 per year (p<0.001).

Most respondents (60.6%, 606) believed that human smallpox cases had occurred in the past 5 years and that effective medical treatment existed for smallpox (Table 1). The likelihood of contracting smallpox by working in close contact with someone with the disease (e.g., in the same office) was considered low by 157 (15.7%), medium by 163 (16.3%), and high by 419 (41.9%); 261 (26.1%) did not know and 1 (0.1%) did not answer.

**Table 1:** Responses to questions on general knowledge of smallpox, Australia, 2004

In the past 5 years, do you think…	Yes, n (%)	No, n (%)	Do not know, n (%)	No answer, n (%)
Human cases of smallpox have occurred in Australia?	127 (12.7)	538 (53.7)	336 (33.6)	0
Human cases of smallpox have occurred somewhere in the world?	606 (60.6)	233 (23.3)	161 (16.1)	1 (0.1)
An effective medical treatment exists for smallpox?	524 (52.3)	114 (11.4)	363 (36.3)	0

A total of 583 (58.2%) respondents stated that they had been vaccinated against smallpox; 346 (34.6%) indicated no prior vaccination against smallpox, 71 (7.1%) did not know, and 1 (0.1%) did not answer. Among 61 respondents born since 1979, the year that smallpox was eradicated and worldwide childhood vaccination terminated, 32 (52.5%) indicated that they had not been vaccinated against smallpox, 20 (32.8%) reported that they had been vaccinated, and 9 (14.8%) did not know. Of 841 respondents born before 1980, 502 (59.8%) reported that they had been vaccinated against smallpox.

The acceptance of vaccination against smallpox under specific hypothetical scenarios was explored. Vaccination would be accepted as an immediate precautionary measure by 41.7% of respondents, while 42.3%, 48.9%, and 56.3% would accept vaccination if cases were reported somewhere in the world, Australia, or their own community, respectively. Among respondents who did not report previous vaccination, 44.5% would accept vaccination as a precautionary measure (Table 2).

**Table 2:** Responses of 418 persons who did not report being vaccinated against smallpox who would accept vaccination under various hypothetical conditions, Australia, 2004

Would accept vaccination…	Yes, n (%)	No, n (%)	Do not know, n (%)	No answer, n (%)
As a precautionary measure?	186 (44.5)	202 (48.3)	15 (3.6)	15 (3.6)
If cases were reported in the world?	193 (46.2)	199 (47.6)	11 (2.6)	15 (3.6)
If cases were reported in Australia?	259 (62.0)	132 (31.6)	12 (2.9)	15 (3.6)
If cases were reported in your community?	332 (79.4)	62 (14.8)	9 (2.2)	15 (3.6)

Modeling the readiness to accept vaccination showed that older persons were less likely to accept smallpox vaccination (odds ratio 0.977 per year, p<0.001). Respondents with more education were also less likely to accept vaccination under any scenario (odds ratio 0.845 per education category, p<0.01).

When asked in an open-ended question where they would first seek diagnosis or care if they thought they had contracted smallpox, 591 (59.0%) respondents mentioned their general practitioner (family physician). Hospital emergency departments were indicated by 330 (33.0%), a public health department by 43 (4.3%), and other sources by 18 (1.8%); 16 (1.6%) did not know and 3 (0.3%) did not answer. Overall, 418 (41.8%) indicated a high level of confidence in their physicians' ability to recognize symptoms of smallpox, 291 (29.1%) a medium level of confidence, 177 (17.7%) a low level of confidence, and 42 (4.2%) no confidence; 68 (6.8%) did not know, and 5 (0.5%) did not answer.

## Conclusions

Most Australian adults interviewed in this national survey reported medium-to-high concern about the risk of a bioterrorism attack in Australia (57.4%) and believed that human smallpox cases had occurred in the past 5 years (60.6%). This finding may explain the general willingness to accept vaccination as a precautionary measure in the absence of a bioterrorism event (*7*). This finding is similar to that of a US survey, which indicated a strong community desire for precautionary vaccination against smallpox (*5*). However, the general public is unlikely to be sufficiently informed to balance the risks of a bioterrorism event against the potential for harm from vaccination (*8*). Given that the currently available smallpox vaccine must produce a significant lesion to be considered effective and commonly results in other adverse events, some severe, mass vaccination as an antiterrorism strategy must be epidemiologically justified by a substantial risk (*9**–**11*). Accurate information on smallpox vaccine adverse effects must be made available to the Australian public, although this information may affect acceptance of vaccination, as was documented among potential medical first responders in the United States (*12*).

Participants were unclear about their personal smallpox vaccination status. Although respondents born after smallpox vaccination was stopped in Australia were incorrect if they believed that they had been vaccinated against smallpox, 33% of this group falsely indicated that had been vaccinated. This belief may lead to a false sense of security in the event of an actual bioterrorist attack with smallpox virus.

Despite the desire for precautionary vaccination, only 259 (62%) respondents who believed they were unvaccinated would accept smallpox vaccination if cases were reported in Australia. A false belief that effective medical treatment exists for smallpox, which was held by more than half of the respondents, may influence decisions to accept vaccination in response to locally occurring cases (*13*). Public health authorities have a clear mandate to improve the community's knowledge of smallpox and bioterrorism. These efforts must involve groups, particularly the elderly and those with more education, who appear more unwilling to accept indicated public health measures.

General practitioners emerged as a pivotal group should a bioterrorism event occur in Australia; respondents identified these medical professionals as the preferred source of initial diagnosis and management and expressed a high level of confidence in their ability to correctly diagnose smallpox. This central role for general practitioners in optimizing biopreparedness in Australia has previously been hypothesized (*14*). Whether the community's belief in the ability and skills of general practitioners is justified is unknown, and this aspect clearly warrants investigation (*15*). Specific training courses for general practitioners that heighten their clinical index of suspicion, introduce public health containment and surveillance principles, and emphasize effective communication strategies should be developed in Australia and accredited for continuing professional development.

Findings in this Australian survey are similar to those in a survey in the United States, even though Australia has not experienced a bioterrorism event. In the US study, a similar proportion of respondents (63%) believed that smallpox cases had occurred in the past 5 years, but a greater proportion would accept precautionary vaccination (61%) and a slightly lower proportion (52%) would go to their own physician for diagnosis and care (*5*). The participation rate of 63% for this survey was similar to that in the US study (65%).

This national survey found that the Australian public holds many inaccurate beliefs about smallpox and smallpox vaccination, and this misinformation could negatively affect response to a bioterrorist event. General practitioners were identified as the primary point of care and should become an important focus of bioterrorism response planning in Australia.

## References

[1] Smallwood RA, Merianos A, Mathews JD. Bioterrorism in Australia. Med J Aust. 2002;176:251–3. 11999254 10.5694/j.1326-5377.2002.tb04400.x

[2] Whitby M, Street AC, Ruff TA, Fenner F. Biological agents as weapons 1: smallpox and botulism. Med J Aust. 2002;176:431–3. 12056996 10.5694/j.1326-5377.2002.tb04486.x

[3] Henderson DA. Bioterrorism as a public health threat. Emerg Infect Dis. 1998;4:488–92. 10.3201/eid0403.980340 9716981 PMC2640310

[4] Smallwood R. Editorial: the risk of anthrax and smallpox in Australia. Commun Dis Intell. 2001;25:188–9. 11806654 10.33321/cdi.2001.25.38

[5] Blendon RJ, DesRoches CM, Benson JM, Herrmann MJ, Taylor-Clark K, Weldon KJ. The public and the smallpox threat. N Engl J Med. 2003;348:426–32. 10.1056/NEJMsa023184 12496352

[6] Alexander DA, Klein S. Biochemical terrorism: too awful to contemplate, too serious to ignore. Br J Psychiatry. 2003;183:491–7. 10.1192/bjp.183.6.491 14645019

[7] Holloway HC, Norwood AE, Fullerton CS, Engel CC, Ursano RJ. The threat of biological weapons: prophylaxis and mitigation of psychological and social consequences. JAMA. 1997;278:425–7. 10.1001/jama.1997.03550050087038 9244335

[8] Pennington H. Smallpox and bioterrorism. Bull World Health Organ. 2003;81:762–7. 14758439 PMC2572332

[9] Jefferson T. Bioterrorism and compulsory vaccination. BMJ. 2004;329:524–5. 10.1136/bmj.329.7465.524 15345604 PMC516092

[10] Centers for Disease Control and Prevention. Update: adverse events following smallpox vaccination—United States, 2003. MMWR Morb Mortal Wkly Rep. 2003;52:278–82. 12729077

[11] Meltzer MI. Risks and benefits of preexposure and postexposure smallpox vaccination. Emerg Infect Dis. 2003;9:1363–70. 14718077 10.3201/eid0911.030369 PMC3035543

[12] Yih WK, Lieu TA, Rego VH, O'Brien MA, Shay DK, Yokoe DS, Attitudes of healthcare workers in U.S. hospitals regarding smallpox vaccination. BMC Public Health. 2003;3:20. 10.1186/1471-2458-3-20 12801426 PMC165598

[13] Henderson DA. Smallpox: clinical and epidemiological features. Emerg Infect Dis. 1999;5:537–9. 10.3201/eid0504.990415 10458961 PMC2627742

[14] Cherry CL, Kainer MA, Ruff TA. Biological weapons preparedness: the role of physicians. Intern Med J. 2003;33:242–53. 10.1046/j.1445-5994.2003.00391.x 12752895

[15] Madeley CR. Diagnosing smallpox in possible bioterrorist attack. Lancet. 2003;361:97–8. 10.1016/S0140-6736(03)12241-6 12531572

